# Reductions in body sway responses to a rhythmic support surface tilt perturbation can be caused by other mechanisms than prediction

**DOI:** 10.1007/s00221-020-05723-z

**Published:** 2020-01-18

**Authors:** Lorenz Assländer, Markus Gruber, Louis-Solal Giboin

**Affiliations:** Human Performance Research Centre, University of Konstanz, Konstanz, Germany

**Keywords:** Standing balance, Postural control, Balance learning, Adaptation, Support surface tilt, Perturbed stance

## Abstract

Studies investigating balance control often use external perturbations to probe the system. These perturbations can be administered as randomized, pseudo-randomized, or predictable sequences. As predictability of a given perturbation can affect balance performance, the way those perturbations are constructed may affect the results of the experiments. In the present study, we hypothesized that subjects are able to adapt to short, rhythmic support surface tilt stimuli, but not to long pseudo-random stimuli. 19 subjects were standing with eyes closed on a servo-controlled platform tilting about the ankle joint axis. Pre and post to the learning intervention, pseudo-random tilt sequences were applied. For the learning phase, a rhythmic and easy-to-memorize 8-s long sequence was applied 75 times, where subjects were instructed to stand as still as possible. Body kinematics were measured and whole body center of mass sway was analyzed. Results showed reduced sway and less forward lean of the body across the learning phase. The sway reductions were similar for stimulus and non-stimulus frequencies. Surprisingly, for the pseudo-random sequences, comparable changes were found from pre- to post-tests. In summary, results confirmed that considerable adaptations exist when exposing subjects to an 8-s long rhythmic perturbation. No indications of predictions of the learning tilt sequence were found, since similar changes were also observed in response to pseudo-random sequences. We conclude that changes in body sway responses following 75 repetitions of an 8-s long rhythmic tilt sequence are due to adaptations in the dynamics of the control mechanism (presumably stiffness).

## Introduction

Standing balance during unpredictable perturbations can be explained by feedback control mechanisms (Peterka 2002). In contrast, balancing in the presence of predictable perturbations could allow subjects to adapt the balance control mechanism and include predictive components. While a number of studies have used predictable perturbations (Corna et al. 1999; Oie et al. 2002; Mergner et al. 2003; Ravaioli et al. 2005; Maurer et al. 2006; Polastri et al. 2012; Sozzi et al. 2016), the adaptations of the balance control mechanism taking place while performing a series of predictable perturbations are not well understood.

### Balance responses to unpredictable perturbations

One method frequently used to identify neural balance control mechanisms applies external perturbations, such as movements of the support surface (Pintelon and Schoukens 2004; van der Kooij et al. 2005). Averaging measured body sway across repetitions of the stimulus sequence reduces the amount of sway variability (random sway) and thereby, extracts the body sway component that is evoked by the stimulus. The relation of stimulus input and body sway evoked by the stimulus provides insight into the dynamics of the control mechanism. Several studies succeeded in reproducing this relation of stimulus and sway response using model (Peterka 2002; Mergner et al. 2003; Assländer et al. 2015; Pasma et al. 2017), and robot simulations (Mergner et al. 2009; Hettich et al. 2014; Pasma et al. 2018). The identified control mechanisms are solely based on delayed sensory feedback and do not contain any predictive components. The lack of predictive contributions is in agreement with the virtually unpredictable properties of the stimulus sequences used in most studies including a modeling approach (Peterka 2002). In other words, since the stimuli cannot be predicted, functionally relevant predictions cannot contribute to balance control. Identified models contain only a few parameters that determine the dynamics of the system. Importantly, these parameters are assumed to be time invariant, i.e. the balance control mechanism is assumed to not change across time.

### Potential adaptations of the control mechanism during predictable perturbations

When standing on a platform that moves with a predictable sequence, the balance control mechanism could adapt in two ways to enhance stability and reduce body sway (Van Ooteghem et al. 2008). One potential adaptation could be a change in the control dynamics, such as changes in sensory reweighting or in the feedback gain (the amount of torque generated per deviation from a desired position). Such adaptations would be non-sequence specific in the sense that the exact shape of the perturbation is not taken into account. Such changes in control dynamics can usually be reproduced by changes in model parameters (sensory weights, feedback gain, etc.). The second adaptation when using a predictable perturbation could be an explicit prediction of the tilt sequence. The implementation of this explicit prediction would be induced by learning processes (Horak et al. 1997). The adaptation would then be sequence specific. According to previous work related to the specificity of balance training, such a specific prediction should not affect the balance control mechanism in other perturbation sequences (Giboin et al. 2015, 2019).

One specific control model proposed by Mergner and colleagues suggests that the central nervous system estimates external perturbations to maintain balance (Mergner et al. 2003; Mergner 2010; Assländer et al. 2015). These estimators are in specific feedback loops that integrate sensory information of multiple sensory systems. Importantly, these estimates have a long time delay, undershoot the actual perturbation (gain < 1), and have a limited sensitivity (implemented as a non-linear threshold mechanism). Mergner (2010) suggested that external perturbations could be learned, where a learned (predicted) internal representation of the perturbation substitutes the sensory reconstruction of this perturbation. The rationale is that the internal representation is superior to the sensory feedback, having reduced time delay, less undershooting and higher sensitivity. Thus, the balance control mechanism would benefit in substituting sensory cues with a prediction, and thereby improve time delay and quality of the compensation signal. The existence of such a mechanism would suggest sequence-specific adaptations when exposed to a predictable perturbation sequence. However, the existence of such a mechanism has not been experimentally confirmed.

### Balance responses to predictable perturbations

Several studies used perturbation sequences that can easily be predicted (Corna et al. 1999; Oie et al. 2002; Mergner et al. 2003; Ravaioli et al. 2005; Maurer et al. 2006; Polastri et al. 2012; Sozzi et al. 2016). The most common example are sinusoidal stimuli. Many of these studies did not report whether or not systematic changes in sway responses occurred across time or when repeatedly applying these stimuli. Furthermore, the first cycles or even trials are usually discarded and familiarization trials are used to avoid initial startles and adaptations. Only a few studies explicitly addressed the adaptation of the balance control mechanism to predictable perturbations. Dietz et al. (1993) used sinusoidal platform translations and induced sudden changes in stimulus frequency. Subjects required 3–4 cycles for the transition into a new steady state of body sway. The authors discussed several mechanisms including a change in the prediction of the stimulus. However, control mechanisms show a transition period when suddenly changing the stimulus, even without predictive contributions (Assländer and Peterka 2014, 2016). Therefore, the results of Dietz et al. (1993) do not provide sufficient information to allow for inferences on predictive contributions.

To the best of our knowledge, only one group used a classic learning paradigm to investigate changes in body sway responses during repeated perturbation sequences. Van Ootegehem et al. (2008, 2009, 2010) used an implicit learning paradigm where subjects were exposed to support surface translation sequences. The sequences were constructed using sinusoidal stimuli with varying stimulus amplitudes. The amplitude modulations were randomly changing for the first and last 15 s of each trial and contained a 15-s long sequence in between that maintained the same tilt sequence across trial repetitions. Subjects showed a reduction in stimulus evoked sway across learning trials, increasingly moving with the platform. Furthermore, a continuous change in phase, i.e. in the temporal relation between stimulus and body sway response, was found across all learning trials. No difference in adaptations between the randomly changing and the repeated component was found. Based on this finding, the authors concluded that the adaptation was non-sequence specific. Using a similar setup, the same authors also showed that adaptations to sequences that were repeated within (three identical 15-s sequences per trial) and across trials did not differ from exposing subjects to random sequences (Van Ooteghem et al. 2010). Similarly, during continuous sinusoidal support translations, a decrease in body sway relative to the platform was found across time (Schmid et al. 2011; Sozzi et al. 2016). In summary, some studies using predictable perturbation sequences found changes in sway responses across sequence repetitions. Also, evidence was found that the adaptations were non-sequence specific. However, the effect was only observed in support surface translations and remains scarce overall.

### Support surface translation vs support surface tilt stimuli

All previous studies addressing the role of predictive mechanisms in perturbed stance used support surface translations. Support surface translations require subjects to shift their body center of mass to move with the platform. Otherwise, the base of support might move away from the projection of the center of mass, resulting in a fall. Using predictable (e.g. sinusoidal) support surface translations allows for two contradictory adaptations: (1) exploit the knowledge, that the support is coming back and keep the body center of mass stable in space or (2) exploit the knowledge where the platform will be moving to shift the body in advance towards the new position. Both strategies have been observed in humans, depending on stimulus frequency and visual condition (Corna et al. 1999; Buchanan and Horak 1999; Nardone et al. 2000). This ambiguity increases the complexity when investigating control dynamics and potential predictive components. Support surface tilts are advantageous, since the task of maintaining the body center of mass upright in space is unambiguous, irrespective of the sequence and visual condition.

### Aim of the study

Earlier studies investigating adaptations to perturbation sequences used support surface translations and implicit learning paradigms only. In the current study, we tested whether sequence-specific adaptations can be observed during an explicit learning task using support surface tilt stimuli. We used virtually unpredictable pseudo-random sequences to characterize the balance control mechanism of subjects before and after a sequence-specific learning session. The learning session consisted of 75 repetitions of an 8-s long rhythmic and predictable surface tilt perturbation. We expected a sequence-specific adaptation, but no transfer to the pre- and post-measurements of the pseudo-random trials. Two hypotheses were tested: (1) body sway in response to an 8-s long predictable stimulus is reduced across 75 repetitions of the stimulus. (2) Responses to pseudo-random stimulus conditions do not change from pre- to post-tests. This study is the first to test adaptations of the balance control mechanism to repeated rhythmic support surface tilt stimuli and the first to test adaptations to repeated perturbation sequences using an explicit learning paradigm.

## Methods

### Subjects

Twelve male and 7 female subjects (aged 24.8 ± 3.8 years, height 173 ± 11 cm, mass 66.8 ± 11.2 kg) participated in the experiments. Prior to the experiments, subjects gave written informed consent. All procedures were performed in agreement with the ethics standards of the University of Konstanz ethics board and with the Declaration of Helsinki in its latest revision.

### Apparatus

During experiments, subjects stood on a custom-made, servo-controlled platform that was commanded to tilt toes-up/down, with the center of rotation approximately at the ankle joint axis. Body sway was measured using sway rods, which rotated about attached potentiometers, and were guided by small hooks located at hip and shoulder level of the subjects. Anterior–posterior sway resulted in angular displacements of the sway rods, which were recorded via the potentiometers. In addition, the device contained a force sensor, measuring the forces transmitted between motor and tilt board. During static conditions (no platform tilt), the force cues were used to assess center of pressure shifts, which were used in a calibration routine to calculate body center of mass (COM) sway (see below).

### Calibration routine

Angular whole body center of mass sway was used for all further analyses. To obtain COM sway from the sway rod data, anterior–posterior translations of the hooks guiding the sway rods were calculated from the potentiometer outputs using the hook heights, hook distances, and trigonometric calculations. We used a two-segment approximation for the COM calculations, where subjects were instructed to hold the arms crossed in front of the chest during all trials. Before starting experiments, subjects were asked to perform very slow tilt movements in the ankle and hip joints for 2 min on the static platform. The center of pressure position is equal to the vertical projection of the body center of mass in static conditions (Brenière 1996). Thus, for this quasi-static condition, a linear regression between center of pressure data and hip and shoulder translations provides calibration factors that allow to calculate the center of mass translation during dynamic trials (Peterka 2002; Assländer and Peterka 2014). Angular COM sway was calculated using the COM translation obtained from the calibration routine and the COM height obtained from anthropometric tables (Winter 2009).

### Stimuli

Two different stimuli were used during the experiments: (1) a pseudo-random ternary sequence used to identify the balance control mechanism before and after the learning phase and (2) a short, rhythmic stimulus for the learning phase.

One pseudo-random cycle was 20-s long and was constructed from 81 velocity steps, which could be either + v, 0, or − v, where three different stimuli with velocities of 0.44°/s, 0.89°/s, and 1.78°/s were used. The velocities were chosen to result in integrated (position) signals with peak-to-peak amplitudes of 1° (pp1), 2° (pp2), and 4° (pp4). The final sequences contained 18 consecutive cycles, resulting in 3 360-s long sequences. In addition to these three stimuli, a 120 s warm-up sequence was created, consisting of two cycles from each amplitude. A detailed description of the construction of pseudo-random ternary sequences can be found in Davies (1970) and Peterka (2002).

The sequence used for the learning trials was an 8-s long superposition of two sine waves at 1 and 1.25 Hz. The sequence contained integer multiples of the sine waves (8 × 1 Hz and 10 × 1.25 Hz). The superposition resulted in a short and rhythmic waxing and waning behavior, which was designed to be memorable and predictable (see also “Discussion” of the predictability). Starting phase, amplitude and offset of both sine components were chosen, such that the sequence had a toes-down bias (maximum tilt 5.3° toes-down and 2.6° toes-up), which was chosen to avoid passive stretch of the calf muscles.

### Instructions for learning sequence and feedback

For the learning phase, subjects received written instructions describing the task to minimize tester influence. The task was to stand as still as possible and to minimize both, dynamic sway and drift. Drift was explained as the difference between starting and end positions in anterior–posterior direction. Also, subjects were provided with the information that actively following the tilt sequence may help to minimize sway evoked by the stimulus. Each learning sequence was started with the command ‘prepare—and go’. In the short breaks (approx. 10 s) in between the 8-s long individual learning sequences, subjects were provided with a feedback score. The feedback score was composed of a sway and a drift component, where higher numbers indicated more sway and/or drift. If one component dominated the feedback (> 70% of the score), the dominant component was provided in addition to the score. The verbal feedback was (translated from German): ‘Your feedback score was *SCORE.* (Dominantly Sway/Drift)’.

The feedback score was calculated after each learning sequence. The drift (*s*) was calculated from the regression slope across the recorded body sway trajectory. Sway from the sum across sway amplitudes obtained from a Fourier transform (*y*) between 0.0125 and 2 Hz. Both scores were scaled, such that the feedback score was typically in the range of 100–500. The feedback was calculated as

$${\text{fb}} = \mathop \sum \limits_{k = 0.0125}^{2} \left| {y\left( k \right)} \right|\; \times \;100 + \left| s \right|\; \times \;1000$$

### Procedures

**Fig. 1:**
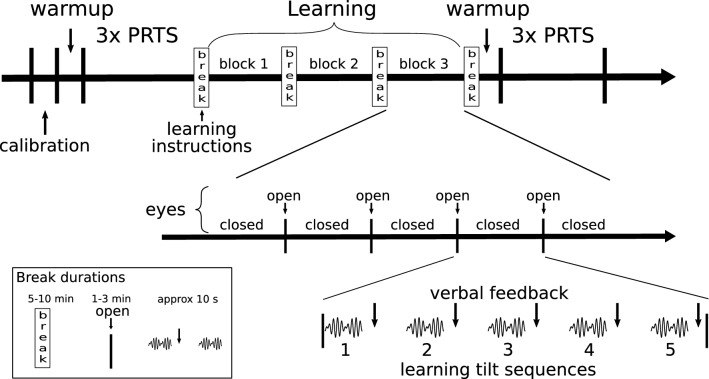
Schematic display of the experimental protocol. After the calibration routine, a warm-up trial familiarized subjects with experimental setup and stimuli. The following three pseudo-random sequences were applied in random order of amplitudes. During warm-up and pseudo-random trials, subjects had their eyes closed, wore noise-canceling headphones, and listened to non-rhythmic audio books to minimize auditory orientation cues and distract from the balancing task. Before the learning trials, subjects were taken out of the experiments, asked to sit, and were provided with written instructions for the learning sequence. Following, the learning sequences were started, where the sequence was always run five consecutive times, while subjects were asked to keep their eyes closed providing feedback in between trials. After five trials subjects were allowed to briefly open their eyes, before continuing with the next block of five trials. After 5 blocks (25 trials), subjects were asked to sit and have a longer break. After a total of 75 trials, the warm-up and the 3 longer pseudo-random trials were repeated as a post-measurement

After providing written informed consent, anthropometric measures for the subjects’ foot placement on the platform and the calculation of the COM position were obtained. Hooks for the sway rods were attached using Velcro belts. Subjects’ feet were placed such that the ankle joint axis was aligned with the platforms’ axis of rotation. Only anterior–posterior position was controlled, while subjects were free to choose stance width and medial/lateral rotation of the feet. After foot placement, subjects were asked to perform the 120-s long calibration routine. The protocol following the calibration routine is shown and described in Fig. 1.

### Data analyses

**Fig. 2:**
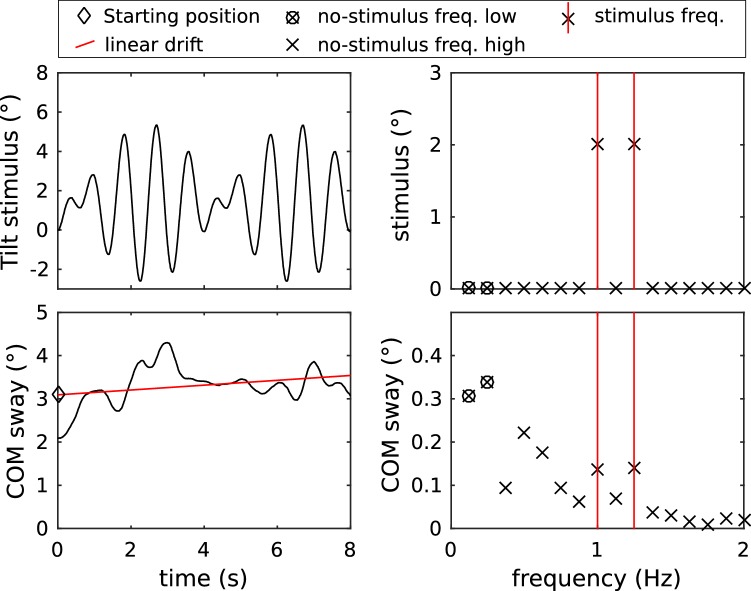
Platform tilt sequence used for the learning trials and representative COM sway during one trial (left) with the corresponding amplitude spectra (right). Vertical red lines indicate the stimulus frequencies. Horizontal red line in the COM sway is the linear regression used to determine the starting position (intercept) and linear drift. In the body sway response, the two smallest frequencies (circles) usually dominated the COM sway spectra and were analyzed as a separate parameter

Figure 2 shows the learning stimulus (top, left) and the COM sway during one individual sequence (bottom, left). For each sequence, a linear regression was calculated for the COM sway trajectory (indicated in red). Offset and slope were subtracted from the sway trajectory, before calculating the Fourier transform. The Fourier spectra were scaled, such that amplitudes represent those of sine waves in the time domain (Fig. 2 top and bottom right, respectively). Several parameters from these calculations were used for the analysis of the adaptations across stimulus repetitions:

1. Full-spectrum PSD (power spectral density)
2. Absolute linear drift (absolute value of the regression slope)
3. Feedback (as calculated above)
4. Stimulus frequency PSD
5. No stimulus, low-frequency PSD (sum of sway response at frequencies 0.125 and 0.250 Hz)
6. No stimulus, high-frequency PSD (sum of all frequencies but stimulus and low-frequency)
7. Starting position (intercept of the regression line)
8. Sway response gain (ratio of body sway to stimulus amplitude at stimulus frequencies)
9. Sway response phase (temporal relation of body sway to stimulus at stimulus frequencies)

Since parameters were calculated for each learning sequence, a series of 75 points was obtained for each of the 6 parameters and each subject. Changes of parameters across 75 repetitions were statistically analyzed (see below).

A detailed description of the analysis of the body sway responses to the tilt stimuli with pseudo-random ternary sequences can be found elsewhere (Peterka 2002; Peterka et al. 2018). In brief, the first cycle of pseudo-random responses was discarded to avoid transient responses. All remaining cycles within each stimulus amplitude and pre/post-condition were averaged across all subjects and stimulus repetitions to obtain time domain sway responses to the stimulus. Furthermore, the Fourier transform of measured stimulus and measured body sway were calculated for each cycle. Calculating the ratio between body sway and stimulus spectra and averaging across all cycle repetitions provides frequency response functions (FRF). A typical representation of these complex-valued functions are Bode diagrams. The absolute value of the FRF (gain) gives the amplitude ratio between body sway response and surface tilt stimulus across frequency. The inverse tangent of the ratio between real and complex component of the FRF averaged across all cycles gives the temporal relation between response and stimulus across frequency (phase). Finally, coherence was calculated, providing a measure of the relation between sway components correlated and uncorrelated with the stimulus sequence. Coherence was calculated from the squared cross-power spectrum between stimulus and response divided by the product of the tilt stimulus and body sway power spectra, where each component was averaged across cycles.

### Statistics

95% confidence bounds for the time domain and frequency domain parameters described above were obtained using bootstrap methods (Zoubir and Boashash 1998). 323 cycles were randomly drawn with replacement from the measured 323 cycles (18–1 cycles × 19 subjects). Thus, in the new dataset, some of the measured cycles are contained multiple times, some are not contained at all. Mean sway responses, as well as frequency response and coherence functions were calculated from the re-sampled dataset. The procedure was repeated 400 times (400 bootstrap samples), where each new dataset was different from the previous one. For each parameter, and condition, the 400 bootstrap samples were ordered in descending order. The 10th and 390th samples (2.5% and 97.5% of 400 samples) were then used as lower and upper confidence limits, respectively.

For the learning period, we were interested in the variation across sequence repetitions for all parameters extracted from the sway response to the learning sequence. For this, we used Bayesian linear mixed models with the R package brms (Brükner 2017). We used a model with the number of performed sequences as a population-level effect (seq effect) and subjects as group-level effect (i.e. random effect). We maximized the error structure to limit type I error (random intercept and slope by subject; Barr et al. 2013). The model used was as follows: dependent variable ~ seq + (seq|subject). We used weakly informative priors (normal distribution with a mean of 0 and a standard deviation of 10 for the beta and a Half Cauchy distribution with a mean of 0 and standard deviation of 2 for the group-level standard deviation). We used 4 MCMC with 4000 iterations each (including a warm-up of 2000 iterations), and verified that each chain converged correctly. Note that we subtracted one to the number of performed sequences, so the first sequence was zero and not one and, therefore, the intercept output from the model was not extrapolated. On top of the population estimate of the intercept and the slope, the model calculated the correlation coefficient between the intercept and the slope at group level (i.e. at subject level).

## Results

### General characteristics of body sway responses to the PRTS stimuli

**Fig. 3:**
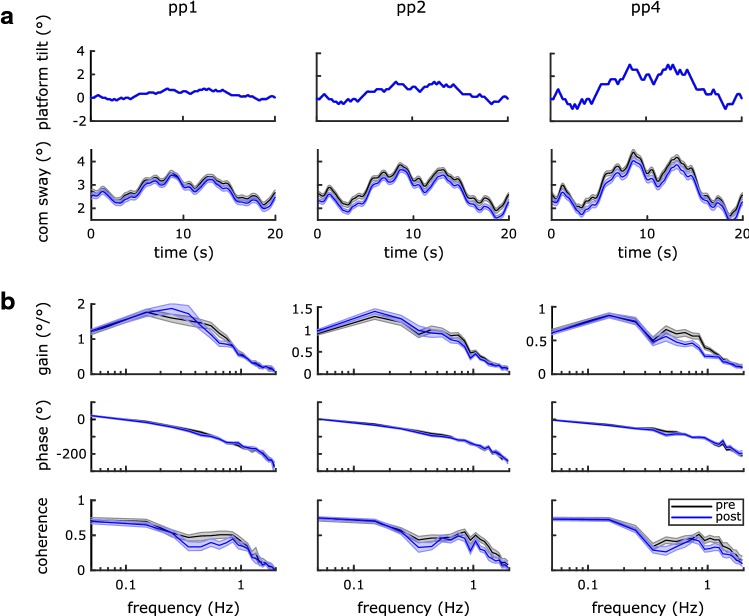
Pseudo-random stimulus and sway responses for pre- and post-measurements in the time domain (**a**) and in the frequency domain (**b**) expressed as amplitude ratio (gain), temporal relation between response and stimulus (phase), and a measure for the sway response to sway variability ratio (coherence). Each stimulus amplitude (peak-to-peak 1, 2, and 4°) is shown in a different column

Figure 3 shows the averaged body sway responses to the pseudo-random platform movement and 95% confidence intervals. The first and second rows show the stimulus and the averaged body sway in the time domain. Sway responses followed the general shape of the stimulus across all stimulus conditions. Subjects were swaying about an offset of 2–3° forward lean. A difference of mean body lean between stimulus amplitudes was found, which was expected since the stimulus had a toes-down bias and subjects tend to align with the platform orientation. Gain, Phase and Coherence across frequencies, shown in Fig. 3a, provide a more detailed analysis of body sway. Gain is the ratio of body sway response to stimulus amplitudes across frequency. A gain of one indicates that body sway and platform tilt amplitudes are identical. A gain smaller/larger than one indicates smaller/larger body sway as compared to stimulus amplitudes at a given frequency. Gain curves showed maxima between 0.1 and 0.3 Hz and decreased towards higher and lower frequencies. Across stimulus amplitudes, gain values decreased with an increase in stimulus amplitude (note the different *y*-axis scales). Thus, the increase in body sway was less than the increase in stimulus amplitude. Phase is a measure of the temporal relation between body sway response and stimulus, where a phase of zero indicates synchronous sway and 180° indicates that body and platform sway are in counter phase. Phase curves showed a small phase lead below 0.1 Hz and an increasing phase lag towards higher frequencies. The phase lag diverged towards higher frequencies, where a smaller phase lag was found for larger stimulus amplitudes. Coherence provides a measure of the contribution of random body sway and sway in response to the tilt stimulus. Coherence can take values between one (only stimulus related body sway) and zero (only random body sway, not related to the stimulus). For all conditions, coherence was about 0.7–0.8 in the frequency range below 0.1 Hz. Coherence decreased between 0.1–0.3 Hz, showing a plateau between approximately 0.4 and 1 Hz and a further decrease at frequencies above 1 Hz. The above-described sway response characteristics for these kind of support surface tilt stimuli are well known and in full agreement with similar studies (Peterka 2002; Assländer et al. 2015; Pasma et al. 2016).

### Differences across pre- and post-conditions

Sway responses in the time domain looked very similar in pre- and post-conditions. However, in post-conditions, the body sway response was shifted towards a more upright body position. Comparison of the mean body lean showed a significant difference between pre- and post-trials for all three amplitudes (two-way repeated measures ANOVA; *p* < 0.001; mean body lean pp1: pre 2.9 ± 1.0; post 2.7 ± 1.0; pp2: pre 3.0 ± 1.0; post 2.7 ± 0.9; pp4: pre 3.1 ± 0.9; post 2.9 ± 1.1). Gain curves did not show consistent differences between pre- and post-conditions below 0.5 Hz and above 1 Hz. In between, gain was smaller in post-conditions as compared to pre-conditions where confidence bounds showed little or no overlap, indicating reduced sway response amplitudes in this frequency range. The phase below 0.7 Hz was smaller in post-conditions and showed a distinct crossing between pre- and post-curves at this frequency. Below 0.7 Hz phase was larger for the post-conditions. Coherence showed no systematic differences above 0.3 Hz. Below 0.3 Hz, coherence had an overall similar shape as in the pre-conditions, but was consistently smaller in the post-condition. All above-described differences between pre- and post-conditions were consistent across stimulus amplitudes.

In summary, the main differences in the post as compared to the pre-conditions were (1) a more upright position (less body lean), (2) reduced sway response amplitudes (gain) between 0.5 and 1 Hz, (3) reduced phase at frequencies below 0.7 Hz and higher phase values at frequencies above 0.7 Hz, and (4) a reduced coherence at frequencies below 0.3 Hz.

**Fig. 4:**
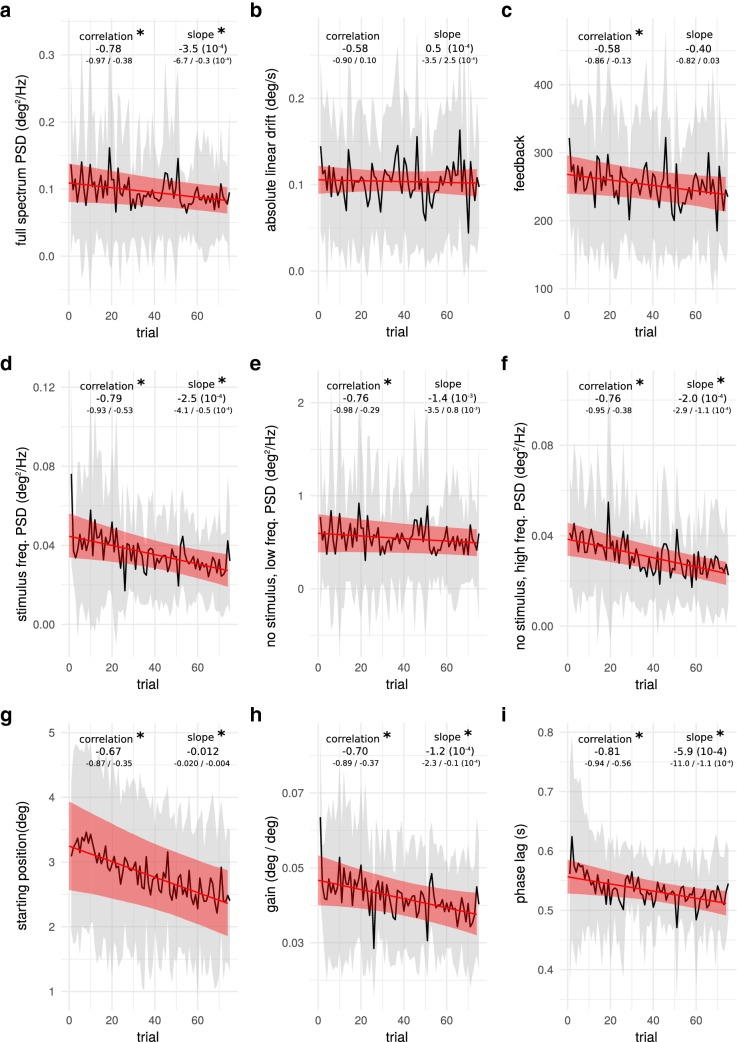
Learning sequence parameters as defined in Fig. 2 across the 75 learning trials. The continuous black line corresponds to the mean parameter value at each trial and the grey area to the standard deviation across all subjects. The red continuous line is the mean estimate from the Bayesian linear mixed model, and the red area its upper and lower 95% credible interval bounds. Correlation and model slope of the Bayesian model and 95% upper and lower credible interval boundaries are given within each plot (intercept is omitted for simplicity), significant differences from zero are indicated with asterisks (95% credible interval that does not frame 0). Correlation represents the relation of intercept and slope at subject level (correlation coefficient of random effects). Statistical significance of correlations and slopes are indicated with asterisks. **a**–**c** show the overall sway as power spectral density (PSD) and drift, as well as the composite score of sway and drift that was used as feedback. **d**–**f**, **h**, **i** provides a more detailed analysis of the sway reduction. **i** shows the starting position of a subject at the begining of the rhythmic stimuli. The parameters displayed in each of the figures are explained in Fig. 2.

Figure 4 shows the parameters extracted from the sway responses during the learning phase across the 75 repetitions of the learning sequence. Feedback and linear drift showed no significant reduction across sequence repetitions. Overall body sway showed a significant reduction across the 75 repetitions of about 30% of the initial sway amplitudes. Sway power at the stimulus frequencies (Fig. 4d) showed a significant decrease across stimulus repetitions (overall 33% reduction). In addition to the systematic reduction across repetitions, a very strong reduction of sway response amplitudes within the first three sequence repetitions was observed. The lowest two frequencies (0.125 Hz and 0.250 Hz) showed the largest sway amplitudes. Since these frequencies dominated the overall spectrum and would have masked potential changes at higher frequencies, sway at these two frequencies were analyzed separately (Fig. 4e). Sway power at these low frequencies showed no significant change during the learning phase. In contrast, random sway at all other non-stimulus frequencies (Fig. 4f) showed a significant reduction of similar magnitude as observed for the stimulus frequencies. In addition to the analysis of the drift and sway amplitudes at different spectral ranges, also the starting position of subjects was analyzed. Starting position was obtained from the intercept of the linear regression calculated for each trial (see Fig. 2 bottom/right). The starting position showed a strong reduction across sequence repetitions. Subjects were leaning approximately 3.3° forward at the beginning and only about 2.4° at the end of the learning phase. A very similar result was obtained when taking the actual starting position (measured body lean at the beginning of the learning sequence; not shown). Sway responses at the stimulus frequencies were in addition analyzed in terms of gain and phase (compare analysis of the pseudo-random stimuli). While the information content of gain is similar as that of the sway power at these frequencies, phase provides in addition insight into the relative timing of support surface tilt and body sway and its development throughout the learning phase. Gain (Fig. 4h) showed a reduction across stimulus repetitions, similar to sway power (Fig. 4d). Phase also showed a significant reduction during the learning phase (Fig. 4i). One additional advantage of the display of body sway at stimulus frequencies in terms of gain and phase is that it can be directly compared to the sway responses to the pseudo-random trials (see “Discussion”).

## Discussion

In this study, we investigated changes in sway responses across 75 repetitions of a rhythmic support surface tilt sequence. In agreement with our first hypothesis, results showed a systematic reduction in body sway throughout the learning phase (see Fig. 4a). However, in contrast to our second hypothesis, systematic changes in body sway responses after the learning phase were also found in the sway responses to the pseudo-random stimuli applied pre and post to the learning phase. Thus, our results show clear adaptations of the balance control mechanism, but no indication of sequence-specific adaptations that could have included predictions. Rather, it appears that subjects adapted the dynamics of the feedback mechanism, such that sway responses to the tilt perturbations were reduced. Further details of our findings and potential adaptations of the control mechanism are discussed below.

### Similarity of changes during learning and pseudo-random stimuli

Since sway response changes were found in both, the learning phase and the pre- and post-pseudo-random tests, the question arises, whether the adaptations underlying both changes are the same. Three parameters can be directly compared between pre-to-post-changes and the learning phase. The starting position was reduced by about 0.7° throughout the learning phase. Since no significant change in drift was found, the starting position can be qualitatively compared to the average body lean during pre–post-pseudo-random tests. Average body lean reduced by 0.2–0.3° from pre- to post-tests, which is a little less than half of that during the learning phase. Comparison of changes in gain and phase showed very similar changes during the learning phase and in pre-to-post comparisons. Gain values were generally smaller during the learning stimulus as compared to the pseudo-random stimuli. This can be expected, since the stimulus amplitudes were much larger in the learning stimulus and larger stimulus amplitudes are known to be associated with smaller gain values. Despite this difference, similar changes in the control mechanism result in similar changes in gain values at a given frequency. Phase values are higher in post-measurements at the frequencies used in the learning stimulus (1 and 1.25 Hz), where higher phase indicates less phase lag. The reduced phase lag corresponded with the decreased phase lag across learning trials. In summary, all directly comparable parameters show similar changes in learning and pre–post-pseudo-random tests. The similarity of the changes across these three parameters supports the assumption that the main adaptations were general changes in the control dynamics.

### Did prediction contribute to the changes?

Humans use predictions to counteract expected perturbations. The most prominent examples are anticipatory postural adjustments, where subjects change body position in expectation of an external or internal perturbation. This notion led us to assume a prediction of the predictable stimulus sequence. The above-discussed similarity of body sway changes to the predictable and the pseudo-random stimulus suggest that predictions are not required to explain the data. However, our data do not exclude the contribution of predictions.

One prerequisite for prediction is the predictive nature of the stimulus. Several aspects may have compromised this assumption. One is the variability in the sensory cues that are used to construct an internal representation of the stimulus. Since there is no sensory receptor directly encoding support surface tilt, a wide range of proprioceptive and vestibular receptor information needs to be integrated to construct an internal representation of the stimulus. This problem is also present in other learning paradigms, but more relevant in the current study, since the estimation of the sequence depends on the ability to estimate body orientation in space from available vestibular cues. The resulting internal reconstruction, therefore, always contains considerable variability, which may reduce the ability to learn the sequence.

Another aspect is that the stimulus may have been to complex for subjects to memorize. Subjectively, however, the rhythmic feature appeared easy to memorize.

### Reduced body sway could be caused by changes in the control dynamics

Subjects were standing more upright following the learning intervention. The reduction during the learning phase was about 20% and about 10% in the pseudo-random trials. The change in body lean is approximately proportional to the torque required to counteract gravity that is constantly pulling the body forward. Therefore, the reduction in lean is associated with a proportional reduction in torque generated by the calf muscles. Since the muscle–tendon complex and the neural feedback mechanisms are non-linear, such a change could have affected the dynamics of the control mechanism. For example, tendon stiffness decreases with decreasing force. Due to this and similar effects, the more upright position could be associated with reduced stiffness.

The changes observed in the frequency response functions are also in agreement with a reduction in the stiffness of the active feedback mechanism. Especially, the reduced gain found at higher frequencies was reported to be related with smaller stiffness parameters in the control dynamics (compare e.g. Fig. 2 in Pasma et al. 2017). Reduced stiffness during toes-up or toes-down tilts of the platform would result in less sway, which is in full agreement with the observed changes in pre–post tests and during the learning phase.

### Rapid reduction in sway power at stimulus frequency and gain after the very first trial

Body sway responses to the very first application of the learning stimulus showed a 65% larger sway amplitude as expected from the general trend of sway response amplitudes (Fig. 4d and g). Notable, the large evoked sway was not associated with a change in body lean. Thus, other changes in the control mechanism might have occurred after the very first trial. The learning sequence had a much larger stimulus amplitude as compared to the pseudo-random trials and subjects were completely naive to the learning stimulus. Thus, the difference could be caused by startles at the beginning of the stimulus. Another explanation could be a very sudden adaptation of the control mechanism through either a reduction in the stiffness of the system or a sudden reweighting, reducing the reliance on proprioceptive cues.

### Limitations

The subjects participating in this study were naive to the protocol and had previously not experienced support surface tilt stimuli in an experimental setup. The pre-to-post-changes and also the change across the learning period could, therefore, be an adaptation of the control dynamics related to the familiarization with the experiment rather than an effect of the learning task. Previous studies using support surface tilt stimuli did not report familiarization effects within one experimental session. However, to the best of our knowledge, no study systematically investigated familiarization effects in such balance experiments. Thus, an important limitation of our study design is that the results do not allow to separate effects from the learning task, where subjects tried to stand as still as possible during the predictable learning stimulus, and adaptations due to a general familiarization with the stimulus and setup.

The feedback score that was verbally provided after each trial did no change significantly across trials. Thus, it is not clear, whether subjects were able to use the feedback to improve performance. Statistically, the drift contribution to the feedback score was not changing across the learning trials, but added variability. The added variability probably prevented the feedback trend to become significant. With the change in feedback score, it might be possible, that subjects had difficulties performing the task they were asked to do (standing as still as possible). However, the systematic changes observed across the learning period showed that some adaptations occurred. Without additional control experiments (e.g. with a similar setup without feedback), no conclusions on the role of the not-changing feedback can be drawn.

## Conclusion

Sway responses to a short and rhythmic support surface tilt sequence change during repeated exposures to the stimulus. Similar changes in sway responses were also found in sway responses to pseudo-random stimuli applied pre and post to the learning trials. Thus, we found no evidence for sequence-specific adaptations and dedicate the observed changes to adaptations of the control dynamics, such as a reduced stiffness.
